# Social connections combat loneliness and promote wellbeing among college students coming out of the COVID-19 pandemic

**DOI:** 10.3389/fpsyg.2025.1529795

**Published:** 2025-02-19

**Authors:** Sabrina Cipolletta, Lucia Ronconi, Silvia Caterina Maria Tomaino

**Affiliations:** Department of General Psychology, University of Padua, Padua, Italy

**Keywords:** college students, loneliness, young adults' mental health, social support, wellbeing

## Abstract

**Introduction:**

The COVID-19 pandemic impacted young people's mental health but we still need to explore this impact in the long term. The aim of this study is to analyze college students' wellbeing and loneliness in relation to the impact of the COVID-19 pandemic, social support, and the frequency of activities carried out with others following the period of strictest restrictions.

**Methods:**

A total of 291 college students enrolled in an Italian University filled in an online questionnaire including measures of anxiety, depression, life satisfaction, loneliness, perceived social support, and the impact of COVID-19 on their quality of life.

**Results:**

The results showed that the greatest impact of the pandemic related to higher levels of loneliness, anxiety, depression and lower satisfaction with life. Conversely, greater social support and frequency of activities carried out with others were associated with lower levels of loneliness and depression, and higher satisfaction with life. Some differences related to gender and being a long-term international student.

**Discussion:**

These results are important to inform interventions to support college students' wellbeing and foster social connections among them.

## 1 Introduction

The COVID-19 pandemic was a worldwide health emergency, drawing attention to its physical, psychological, social and economic consequences, both at the time and also in the long term. Studies conducted 1 year on from the onset of the pandemic have shown that its impact on mental health was on-going (Benke et al., 2022; Bourmistova et al., 2022; Fountoulakis et al., 2022) especially as a consequence of the forced social isolation experienced during this unprecedented and prolonged situation. People experienced psychological suffering as a result of working and studying from home, being apart from loved ones, and being afraid of contracting an infection (Arslan and Allen, 2021; Todorova et al., 2021). A qualitative study carried out in Italy (Tomaino et al., 2021) found that when working from home, many experienced difficulties in staying motivated and productive, while others said they felt lonely and missed out on social opportunities. In addition, they reported a general sense of confusion and uncertainty about the future, and a desire to return to their usual everyday activities.

Such knowledge resulting from exposure to the pandemic has raised awareness of the importance of the role of social support in preventing loneliness and fostering wellbeing (Lampraki et al., 2022; Wang et al., 2018). Indeed, even if perceived social support decreased throughout the pandemic (Li et al., 2021), those who reported high social support had a significantly lower risk of experiencing depression, sleeplessness, anxiety and loneliness (Grey et al., 2020; Ma et al., 2020). Loneliness and social support have an inverse relationship: people who feel socially supported are less subject to loneliness (Matthews et al., 2016; Teo et al., 2013; Zhang and Dong, 2022). Conversely, people who are socially isolated or lack adequate social support are more vulnerable to loneliness and suffer implications in terms of their mental health and wellbeing (Hwang et al., 2020). The buffering model of social support (Cohen and Wills, 1985) attributes the positive association between social support and wellbeing to a process of support protecting persons from potentially adverse effects of stressful events. Similarly, the models of social isolation and its psychological consequences (Hawkley and Cacioppo, 2010) provide a stronger conceptual foundation for interpreting the relationship between loneliness and wellbeing. These models delve into the mechanisms by which loneliness impacts physical and mental health, thereby providing a comprehensive understanding of the interplay between social connectedness and wellbeing.

Previous studies (Beam and Kim, 2020; Glowacz and Schmits, 2020; Houghton et al., 2022) point out that young adults, particularly those between the ages of 18 and 30, have been the group to experience the greatest negative effects during the pandemic (Glowacz and Schmits, 2020; Houghton et al., 2022). Along with a high prevalence of symptoms of anxiety, depression and stress, during the pandemic young adults experienced loneliness (Horigian et al., 2021; Lee et al., 2020; Lisitsa et al., 2020; Zhong et al., 2023). The influence of restrictive measures, particularly social distancing, changed the quantity and frequency of social connections, sometimes leading to a considerable disruption of social networks (Lampraki et al., 2022). The developmental perspective of emerging adulthood (Arnett, 2000) can help to explain why young adults are especially vulnerable to the effects of social disruptions during the pandemic. Emerging adulthood is a time of active exploration in areas such as career paths, relationships, and personal values. Social interactions and peer relationships are critical during this stage, serving as avenues for exploration and support. The pandemic disrupted many opportunities for self-discovery, such as internships, study-abroad programs, social engagements, and early career experience. These disruptions may have delayed or derailed key identity-forming experiences, leaving individuals feeling uncertain and anxious about their futures. The interference with identity exploration, the exacerbation of instability, and the restriction of opportunities for independence and social connection undermined their developmental goals and increased psychological distress.

Recent systematic reviews and metanalyses (Buizza et al., 2022; Carvalho et al., 2022; Elharake et al., 2023; Luo et al., 2021) point out that college students experienced particular challenges in terms of disruption to their social and academic lives, with negative consequences for their mental health (Abbas et al., 2024; Cao et al., 2020; Haikalis et al., 2022; Savarese et al., 2020). The uncertain duration of coping measures and, consequently, of the use of remote learning, was a source of worry in terms of missing social gatherings while leading students to be more concentrated on saving time to complete the degree program (Abbas et al., 2024). Therefore, college students have been considered a population of interest when investigating the psychosocial consequences of the pandemic.

Within the population of college students, some variables must be taken into consideration based on the results of previous studies. First of all, gender differences. Some studies have found that being a female predicted higher anxiety and lower depression symptoms (Wenjuan et al., 2020); however, other studies found no gender differences in this population (Cao et al., 2020; Parola et al., 2020). These contrasting data suggest the usefulness of further exploring gender differences within this population. International students have also been mentioned as being more vulnerable and at risk during such unprecedented times (Firang, 2020; Hari et al., 2021; Kivelä et al., 2022; Mbous et al., 2022). In fact, this population is recognized as facing challenging and novel circumstances, including a new cultural and language environment, new housing, and the absence of familial sources of social support; this frequently results in emotions of loneliness and isolation (Sawir et al., 2008). Cultural adjustment theories (Berry and Sam, 1997) point out that international students face specific challenges, particularly regarding cultural loneliness and acculturation stress. Cultural loneliness is a feeling of isolation that results from being in a foreign cultural or linguistic environment (Sawir et al., 2008); this has been proven to be highly relevant among this population and linked to poorer perceived social support and higher psychological discomfort (Cipolletta et al., 2022). During the pandemic, international students faced additional and specific disruptions such as fear and anxiety related to accessing healthcare in a foreign country as well worrying about the health of family members in their home country (Hari et al., 2021), stigma and discrimination (Chen et al., 2020), the closure of university campuses and other facilities, housing insecurities, together with difficulties with learning and socializing with peers.

Limited amounts of data are available on the effects of reduced contact with peers and social support on college students' wellbeing after the end of the COVID-19 restrictions when college students were slowly engaging with their social environments, albeit with many uncertainties and facing the complexity of a new way of being in relationship with others (Almarashdi, 2024; Bersia et al., 2024; Dingle et al., 2022; Ren et al., 2021). Studying this specific phase provides an important opportunity to identify potential resources and obtain knowledge to inform policy makers and academic staff with relevant information when it comes to creating timely and effective interventions, preventing similar outcomes in the long term.

The aim of the present study was to investigate college students' wellbeing and loneliness in relation to the perceived impact of the COVID-19 pandemic, social support, and the frequency of activities carried out with others in the period of transition from online to in-person educational activities (i.e. during the academic year 2021/2022). Specifically, the hypotheses of this study were:

1. College students would report high levels of loneliness, anxiety and depression;
2. Within the sample some differences would have been related to gender and being international students. Such students would report higher levels of loneliness, anxiety and depression and perceive less social support than local students; furthermore, their quality of life would have been more affected by the COVID-19 pandemic than local students. Given the contrasting results of previous studies, no specific hypotheses were formulated relating to gender differences.
3. Doing activities with others rather than alone during the academic year 2020/2021 would have been associated negatively with measures of anxiety, depression and loneliness, and positively with satisfaction with life;
4. The perceived impact of the COVID-19 pandemic on participants' quality of life would have corresponded with higher levels of loneliness, anxiety, depression and a lower satisfaction with life;
5. Higher social support would have been associated with lower levels of loneliness and depression, and a higher satisfaction with life.

## 2 Material and methods

### 2.1 Participants

A total of 291 students of the University of Padua (Italy) took part in the present study. However, only data from 268 participants were used in the final analysis due to failure to complete at least 90% of the survey on the part of the other respondents. Of the 268 respondents (201 women, 66 men and one non-binary), aged between 18 and 42 years (*M* = 22.86, SD = 3.04), 198 were Italian students and 70 were international students, of whom 84.29% (59) were long-term international students, with the remaining 15.71% (11) being short-term (Erasmus) students. The mean length of stay for international students was 17.16 months (SD = 11.64). Out of the total sample, 49.25% (*n* = 132) reported attending at least 75% of their lessons in-person, while the proportion of students attending mostly online was 25.75% (*n* = 69). Furthermore, 18.66% (*n* = 50) reported receiving professional psychological support at the time of the data collection and 41.42% (*n* = 111) reported having received psychological support at least once in their lifetime. Other characteristics of the sample can be found on Table 1.

**Table 1:** Socio-demographic characteristics of the sample.

	***n***	**%**		***n***	**%**
**Gender**			**Group**		
Male	66	24.63%	Italian students	198	73.88%
Female	201	75%	International students	59	22.02%
Other	1	0.37%	Erasmus students	11	4.10%
Total	268	100%		268	100%
**Provenance (Italian non-residential students)**			**Provenance (International and Erasmus students)**		
North of Italy	73	70.19%	Africa	3	4.29%
Center of Italy	11	10.58%	America	14	20%
South of Italy	20	19.23%	Asia	26	37.14%
			Europe	27	38.57%
Total	104	100%		70	100%
**Living situation**			**Degree attended**		
Apartment with flatmates	135	50.37%	Bachelor's degree	137	51.12%
Living with family	102	38.06%	Master's degree	122	45.52%
Student dormitory	16	5.97%	Postgraduate internship	3	1.12%
Apartment alone	7	2.61%	Short specialization program	3	1.12%
Living with significant other	4	1.49%	PhD	3	1.12%
B&B/Hotel/Hostel	3	1.12%			
Homeless	1	0.37%			
Total	268	100%		268	100%

To determine the necessary sample, two power analyses were conducted using G^*^Power. A first power analysis, to determine the minimum sample size request for a medium size of slope for linear bivariate regression (Slope H1 = 0.15) with a power of 0.80, alpha of 0.05 and test family *t*-test (one tail), indicated the need for at least 270 participants. A second power analysis, to determine the minimum sample size request for a medium effect size for mean differences between two independent groups (Cohen's *d* = 0.50) with a power of 0.80, alpha of 0.05 and test family *t*-test (two tails), indicated at least 64 participants would be necessary in each group.

Participants were recruited by posting notices on university social media (Facebook, Instagram) and by actively involving students' representatives and degree course coordinators. In order to be included in the study, participants had to be at least 18 years of age, and be enrolled full-time in a degree program at the University of Padua, or Erasmus short-term international students. The Ethical Committee for the Psychological Research of the University of Padova approved the study (protocol n. 5112).

### 2.2 Data collection

The participants filled in an online survey composed of a total of 60 items, distributed via Qualtrics (https://www.qualtrics.com), both in Italian and in English, between September 2022 and January 2023. The choice with regard to using an online survey was due to practical and ethical considerations. Conducting the survey via an online platform enabled us to reach a broad audience efficiently while minimizing logistical constraints and ensuring confidentiality and ease of participation, thereby reducing potential barriers to participant engagement. The use of non-probability sampling allowed us to obtain initial insights into the research question, while recognizing its limitations for generalizability.

The survey was divided into two sections and took about 10 min to complete. The first section (15 questions) was created to collect socio-demographic data, as well as data on participation in academic and extracurricular activities for the academic year 2021–2022.

The second part of the survey (45 items) was divided into the following six short self-report questionnaires:

– The ULS-6 (Neto, 1992), which consisted of a short version of the UCLA Loneliness Scale (Russell et al., 1978) to measure loneliness as a general construct. It is composed of six items evaluated on a 4-point scale in a range from 0 to 18, with the same validity as the long version and a good level of reliability (α =0.77). The ULS-6 has never been validated in Italy. In order to include it in the Italian version of our survey, the researchers retrieved the Italian translation of four of the six items from the Italian translation of the ULS-7, conducted by Zammuner (2008); since four of the seven items corresponded to the ULS-6 items, it was possible to partially rely on that translation. The researchers later conducted a back-translation of the two remaining items and conducted a trial to evaluate their comprehensibility; finally, Cronbach's in alpha was measured order to verify the reliability of our translation (α = 0.85).
– The Multidimensional Scale of Perceived Social Support (MSPSS) (Zimet et al., 1988) assesses social support from different sources, and is composed of 12 items and three subscales: Family, Friends and Significant Others. The original scale has a good level of reliability (α = 0.88). Each item of the scale is rated on a 7-point Likert scale going from “very strongly disagree” to “very strongly agree”; each subscale is composed of four items addressing various types of support. The final score ranges between 12 and 84. An Italian version of this instrument was created and validated, showing psychometric properties comparable to the original (Di Fabio and Palazzeschi, 2015).
– The General Anxiety Disorder-7 (GAD-7) is an instrument designed for the screening, assessment and diagnosis of the generalized anxiety disorder (Spitzer et al., 2006). The GAD-7 is composed of seven items evaluated on a 4-point scale with scores ranging from 0 to 21: each item represents one symptom, whose frequency has to be rated by the respondents in reference to the previous 2 weeks. Cut-off scores of 5, 10, and 15 represent mild, moderate, and severe anxiety levels. The GAD-7 reliability is reported to be 0.88 (Johnson et al., 2019).
– The Patient Health Questionnaire-9 (PHQ-9) (Kroenke et al., 2001) was developed to assess depressive symptoms the patient has experienced in the previous 2 weeks. It is composed of nine items evaluated on a 4-point scale. The total PHQ-9 score ranges from 0 to 27, with higher scores indicating more severe symptoms. Cutoff scores of 5, 10, and 15 represent mild, moderate, and severe levels of depression. The PHQ-9 has good construct validity and a reliability between 0.86 and 0.89 (Beard et al., 2016). The Italian version of the GAD-7 and PHQ-9 were recently tested (Bolgeo et al., 2023).
– The Satisfaction with Life Scale (SWLS) (Diener et al., 1985) is a measure of subjective wellbeing and it is composed of five items evaluated on a 7-point scale with a reliability of 0.87, which is similar for the Italian version (Di Fabio and Gori, 2016).
– The COVID-19—Impact on Quality of Life scale (COV19-QoL) (Repišti et al., 2020) is a short measure of perceptions of the impact of the COVID-19 pandemic on quality of life. It is composed of six items evaluated on a 5-point scale, with a reliability between 0.85 and 0.88. This instrument has never been validated in Italy; the researchers conducted a back-translation of the items and a trial to evaluate their comprehensibility in order to be able to include it in the final survey. Finally, Cronbach's alpha was measured in order to verify the reliability of our translation (α = 0.85).

### 2.3 Data analysis

Rstudio software was used for the statistical analysis. Descriptive analysis was conducted on the socio-demographic and questionnaire data. The normality distribution assumption was checked by absolute values of skewness and kurtosis (< 1) and the Shapiro–Wilk test.

The non-parametric Mann–Whitney *U*-test was used for group comparison due to the violation of the normality distribution assumption with regard to several variables and, above all, due to the high numerical imbalance of the groups in terms of gender and for the comparison between local and international students. Bonferroni's correction for multiple significance tests was involved, adjusting the critical significance level of 0.05 by dividing it by the number of statistical tests performed relating to social support, loneliness and mental health (six comparisons, *p* < 0.008). Moreover, the non-parametric Mann–Whitney *U*-test was used for a comparison between long-term and short-term in the international students' subsample.

The Spearman correlation coefficient was used to explore the relationship between social support, the frequency of activities carried out with others, activities carried out alone, of lessons attended in-person, of those attended online, and living with cohabitants, with measures of loneliness, anxiety, depression, life satisfaction and the impact of the COVID-19 pandemic. Bonferroni's correction for multiple significance tests was involved, adjusting the critical significance level of 0.05 by dividing it by the number of statistical tests (11 correlations, *p* < 0.005).

Finally, a path analysis model was tested using the perceived impact of COVID-19 on quality of life and social support as predictors of loneliness, anxiety, depression and life satisfaction. The level of statistical significance for path coefficients was set at = 0.05.

## 3 Results

### 3.1 Students' wellbeing and comparison between groups

Descriptive analyses showed that 40.67% of the sample reported moderate to severe levels of anxiety while 37.69% reported moderate to severe depression. More than half of the sample (53.36%) reported scores between slight and extremely satisfied with life, while social support was also high for 72.39% of the participants. The participants' mean score in the ULS-6 was 8.53 (SD = 4.47) indicating a moderate level of loneliness. The mean score on COV19-QoL was 2.89 (SD = 0.89) on a scale from 1 to 5. Given this overall picture, differences in the scores were analyzed in reference to the socio-demographic characteristics of the sample.

As regards gender differences, women reported a higher level of social support, but also higher levels of anxiety and a higher impact of COVID-19 pandemic on their quality of life compared to men (Table 2). After Bonferroni correction the difference on social support was not significant because the *p*-value was higher than 0.008.

**Table 2:** Differences between male and female students in the scores of the questionnaires.

**Scale**	**Gender**	**Mean**	**Median**	**SD**	**Mann–Whitney *U***	***p*-value**	**Spearman r**
ULS-6	Male	7.82	8.00	4.42	7,483	0.117	0.128
	Female	8.73	9.00	4.46			
MSPSS	Male	62.86	67.00	12.2	7,856	0.024	0.185
	Female	66.83	68.00	10.8			
PHQ-9	Male	8.11	6.50	5.90	7,483	0.117	0.147
	Female	9.52	8.00	6.25			
GAD-7	Male	7.73	6.00	5.69	8,236	0.003	0.242
	Female	9.62	8.00	5.37			
SWLS	Male	20.29	21.00	6.72	5,984	0.306	0.084
	Female	21.30	21.00	5.95			
COV19-QoL	Male	2.64	2.67	0.758	4,935	0.003	0.235
	Female	2.96	3.00	0.921			

Another comparison was conducted between Italian and international students in terms of levels of loneliness, social support, anxiety, depression, life satisfaction and the impact of the COVID-19 pandemic. Italian students reported higher perceived social support than international students; conversely, life satisfaction was higher for international students compared with the Italian group, but none of these comparisons was significant with the correction for alpha error (Table 3).

**Table 3:** Differences between Italian and international students in the measures of interest.

**Scale**	**Group**	**Mean**	**Median**	**SD**	**Mann–Whitney *U***	***p*-value**	**Spearman r**
ULS-6	Italian	8.57	8.50	4.18	7,070	0.801	0.020
	International	8.41	8.50	5.23			
MSPSS	Italian	66.79	69.00	10.66	8,232	0.019	0.188
	International	63.06	64.00	12.52			
PHQ-9	Italian	9.28	8.00	6.03	7,341	0.433	0.063
	International	9.07	7.50	6.82			
GAD-7	Italian	9.25	8.00	5.21	7,366	0.460	0.059
	International	9.04	7.50	6.41			
SWLS	Italian	20.54	20.00	5.82	5,504	0.016	0.194
	International	22.47	22.50	6.69			
COV19-QoL	Italian	2.89	3.00	0.84	6,764	0.963	0.012
	International	2.90	3.00	1.03			

Within the international students group, long-term students compared to short-term students reported significantly higher levels of depression (*U* = 200, *p* = 0.044, *r* = 0.385) and higher impact of the pandemic on their quality of life (*U* = 174, *p* = 0.015, *r* = 0.464) lower social support (*U* = 132, *p* = 0.002, *r* = 0.593) and lower satisfaction with life (*U* = 196, *p* = 0.039, *r* = 0.396).

### 3.2 Relationships between variables

Spearman correlation analysis was conducted to assess the association between social support, the frequency of activities carried out with others, activities carried out alone, lessons attended in-person, lessons attended online, and living with cohabitants, with regard to the measures of loneliness, anxiety, depression, life satisfaction and impact of the COVID-19 pandemic. The results indicated that a higher frequency of activities carried out with others was correlated with higher perceived social support and life satisfaction; a higher frequency of the same activities was also associated with lower levels of loneliness, anxiety and depression, together with a lower perceived negative impact of the pandemic on quality of life. Moreover, a higher frequency of activities carried out alone was associated with lower perceived social support and higher levels of loneliness, as well as symptoms of depression and anxiety. The results are reported in Table 4.

**Table 4:** Spearman correlations between the measures of wellbeing and participants' living conditions and activities.

	**MSPSS**	**ULS-6**	**PHQ-9**	**GAD-7**	**SWLS**	**COV19-QoL**
Frequency of activities with others	0.23^***^	−0.30^***^	−0.27^***^	−0.22^***^	0.25^***^	−0.24^***^
Frequency of activities alone	−0.16^**^	0.18^**^	0.17^**^	0.14^*^	−0.07	0.06
Frequency of lessons in presence	0.03	−0.01	−0.10	−0.11	0.12	−0.06
Frequency of lessons online	−0.04	0.03	0.02	−0.02	−0.07	0.04
Number of cohabitants	−0.09	−0.04	−0.006	−0.001	−0.09	0.03

^*^p < 0.05.

^**^p < 0.01.

^***^p < 0.001.

**Figure 1:**
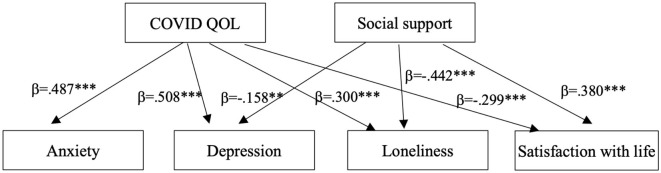
Path analysis of the relationships between the variables. **p <0.01, ***p <0.001.

The path analysis revealed that a greater perceived impact of the pandemic and lower levels of social support were associated with increased loneliness and depression, as well as reduced satisfaction with life. The standardized coefficients (β), indicating the strength and direction of the relationships between the variables, and their statistical significance, are reported in Figure 1. The model has zero degrees of freedom and is therefore saturated, that means it reproduces the observed means, variances, and covariances perfectly because it estimates as many parameters as there are available pieces of information from the data. Saturated models always fit perfectly, with RMSEA = 0.000 and CFI = 1.000, suggesting no discrepancy between the model-implied and observed covariance matrices; thereby R-square values were used to assess the model fit. *R*-square values varied between 0.268 for GAD-7 and 0.378 for ULS-6, thus suggesting that the model explained varying proportions of variance across the dependent variables: 26.8% for GAD-7 (*R*^2^= 0.268), 33.9% for PHQ (*R*^2^ = 0.339), 31.3% for SWLS (*R*^2^ = 0.313), and 37.8% for ULS-6 (*R*^2^ = 0.378). These values indicate that the predictors accounted for a moderate proportion of variance in all outcomes, with the strongest explanatory power observed for loneliness (ULS-6) and the lowest for anxiety symptoms (GAD-7). The results suggest that while the model captures important predictors of these constructs, other factors not included in the model may also contribute to their variance.

## 4 Discussion

This study aimed to explore college students' wellbeing and loneliness in relation to the perceived impact of the COVID-19 pandemic, social support, living conditions and the frequency of activities carried out with others after the strictest restriction phase. The first step was to obtain a picture of the population that participated in the study. The results showed a persisting deterioration of students' wellbeing during the post-pandemic era. Indeed, the participants reported a relatively high incidence of both moderately severe to severe depressive symptoms, and severe anxiety; this is a lower prevalence when compared to those reported by research carried out on young adult populations at the early stages of the pandemic when social isolation was imposed to limit the spread of the virus (Faisal et al., 2022; Liu et al., 2020; Varma et al., 2021). However, a similar incidence was found in an Italian sample at the start of the pandemic (Rossi et al., 2020) and around the same time as this study (Bersia et al., 2024).

The majority of the participants reported the pandemic as having a moderate impact on their quality of life, moderate loneliness, high perceived social support and satisfaction with life. These findings must be contextualized in the period of transition from the strictest phase of the pandemic to a consistent restoration of social and in-person academic activities, thus resulting in contrast with data collected during the first phases of the pandemic when social isolation was imposed to limit the spread of the virus (Elharake et al., 2023; Li et al., 2021; Lisitsa et al., 2020; Senese et al., 2024).

The persisting of poor metal health outcomes in the post-pandemic era can be explained by the transition back to normalcy that might have introduced new stressors, such as: pressure to catch up on academic or career progress, challenges in rebuilding social connections, and adjusting to a “new normal” where expectations may have shifted. These challenges might sustain or exacerbate mental health issues. The lower prevalence of severe depressive and anxiety symptoms compared to the early pandemic stages could reflect an initial overburden on mental health due to social isolation, fear of the unknown, and abrupt lifestyle changes. Over time, individuals and communities may have developed some level of adaptation or resilience, which may explain why current rates, while concerning, are lower than during the early pandemic. The finding of a similar prevalence to those in the Italian sample during the early pandemic and the present suggests that some factors contributing to mental health deterioration may remain constant, such as lingering academic pressures, social reintegration challenges, or economic instability. It is also important to consider potential cultural or systemic factors that may influence mental health responses, including the readiness of the healthcare system, the availability of social support structures, and cultural attitudes toward mental health.

The second research question wanted to explore if there were differences in the students' wellbeing due to gender and to their being international students. In line with previous studies (Cao et al., 2020; Parola et al., 2020; Wenjuan et al., 2020) the results revealed higher anxiety and an increased impact of the pandemic on the quality of life in women than in men. No significant gender differences were found with regard to social support, loneliness, depression or life satisfaction. In the specific case of international students, we expected a more detrimental effect of the pandemic on the quality of life and worse mental health compared to Italian ones. Interestingly, despite the fact that Italian students' levels of perceived social support were higher than those of international students, there was no significant difference in levels of social support, loneliness, anxiety, depression, life satisfaction and the effects of the pandemic on quality of life.

These findings contrast with those of earlier studies (Skromanis et al., 2018), which suggested that international students had poorer mental health and lower wellbeing than local students, but are in line with more recent data collected after the pandemic (Dingle et al., 2022). As Kivelä et al. (2022) suggest, it is possible that local students' mental health has deteriorated more noticeably during the pandemic, leading to outcomes that are comparable with those found among international students. This might be the case with the participants of this study; however, since this study was cross-sectional, it is not reasonable to presume that the mental health of the local sample has also worsened. Another possible explanation could be found in the reported high motivation of international students following a path for personal growth and investment in their future (Milian et al., 2015; Sobkowiak, 2019). This might especially apply to short-term students as the results show that international long-term students reported more severe depressive symptoms and a greater impact of the pandemic on their quality of life, together with a lower level of social support and less satisfaction with life. This result is consistent with those collected before the pandemic in the same population (Cipolletta et al., 2022).

With regard to the third research question, the results of the present study show that participants who reported frequent engagement in activities with others also reported lower levels of loneliness, anxiety and depression, whereas those who mainly carried out activities alone reported the opposite psychological condition. Furthermore, the frequency of the activities carried out with others was characterized by a negative correlation with the perceived impact of the pandemic on the participants' quality of life, underlining that those who reported a higher impact of the pandemic were engaging less frequently in social activities with peers. In contrast, the frequency of the activities carried out with others was positively correlated with perceived social support and life satisfaction, reinforcing the previous associations. These results suggest that a commitment to social relationships promotes wellbeing, as a recent study conducted with the same population confirm (Cipolletta et al., 2024). Previous literature points out that social distancing and changes to the quantity and frequency of social interactions due to the pandemic sometimes led to a significant disruption of social networks (Lampraki et al., 2022) and loneliness (Ernst et al., 2022). Disruptions in social interactions can have a negative impact on mental health (Kim and Cho, 2020; Shen et al., 2022) because there is a link between carrying out activities with others frequently and better psychological wellbeing, rather than simply being engaged in leisure activities.

Finally, the results of the present study support the connection between social support and wellbeing (Cahuas et al., 2023; Clair et al., 2021; Costa et al., 2022; Grey et al., 2020; Lee et al., 2020): in fact, students' perception of social support predicted lower levels of loneliness and depression, and higher satisfaction with life. In contrast to the protective role of social support, the perceived impact of the COVID-19 pandemic on the participants' quality of live was related to higher levels of loneliness, anxiety and depression, and lower satisfaction with life.

The findings of the present study stress the important effect of social interactions with others and social support in promoting mental health and wellbeing. While it is not possible to draw conclusions regarding causal relationships from the nature of our analysis, it is possible to hypothesize that frequent social interactions with others on the part of college students may have reduced their feelings of loneliness, anxiety and depression. However, young people who show similar symptoms might also be less likely to leave their home and interact with others, which would reduce the frequency of social activities. Similar inferences might be made regarding the effects of the pandemic: people reporting a higher impact of the pandemic on their quality of life may have had more distress in their lives since the onset of the pandemic, which may have led them to stay home more frequently. In contrast, people who were able to carry on frequent social activities may have felt less of a difference between their lives prior to and after the pandemic, which could have resulted in a lesser impact of the pandemic on their quality of life. Social support can indeed buffer the effects of stress, as proposed by Cohen and Wills (1985), through mechanisms of emotional validation and practical resource facilitation. By mitigating the emotional and situational impacts of stressors, social support can reduce loneliness and the risk of depression, offering a compelling framework for psychological and social interventions.

### 4.1 Limitations

The present study has some limitations. The first limitation results from its cross-sectional design in that it captures data at a single point in time. As a result, it does not allow for the assessment of causality or the directionality of observed associations. Additionally, this particular design may be subject to temporal bias, as it does not account for changes in variables over time. A longitudinal or experimental design would allow researchers to examine the directionality of these associations. Combining quantitative data with qualitative interviews or behavioral logs of social interactions might help to understand the causal effects of social support on mental health and could provide deeper insights into students' experiences and coping mechanisms.

Another limitation of this study is the potential for selection bias inherent in the use of online surveys. Participants were self-selected, which may have excluded individuals less inclined to engage in online studies. The reliance on self-reported data introduces the possibility of recall bias or reporting inaccuracies, even more as some events (e.g. activities alone or with others) were retrospectively recalled by participants with regard to the previous academic year. Moreover, the number of participants is not representative of the total student population at the University of Padua. Our sample consisted of a disproportionate number of female students (75%), underlining the importance of balancing the sample in future research. All these limitations restrict generalizations and the establishment of causality.

Finally, it must be kept in mind that the international students completed the survey in English that for most of them was a second language. This could introduce a potential source of bias or increase the chances of non-accurate responses. Although international students must have at least a certified B2 level of English to enroll in an international degree program at the University of Padua or to participate in an exchange program, the potential difficulties in understanding and correctly interpreting the questions, could have resulted in the tests' lack of sensitivity to the respondents' cultural diversity and language ability.

## 5 Conclusion

To conclude, the results of the present study suggest that in the post-pandemic era the perceived impact of COVID-19 pandemic on quality of life is still associated with poorer mental health and wellbeing on the part of college students, and social support has a buffering role to play in alleviating such an impact. The participation in activities with others also plays an important role in promoting college students' wellbeing. These findings have significant public health implications in terms of addressing the psychological impact of the pandemic. The association between the perceived pandemic's impact and heightened levels of loneliness, anxiety, and depression highlights the need for targeted mental health interventions. Universities and public health agencies should prioritize resources to address pandemic-induced psychological distress, particularly for young adults who may be experiencing long-term repercussions. Continuous monitoring of students' mental health and wellbeing is essential to ensure the effectiveness of interventions. Implementing regular assessments and feedback mechanisms can help tailor support services to evolving student needs, especially in the face of ongoing or future public health crises. Policymakers should consider the broader societal implications of pandemic-induced mental health challenges for young adults. Investments in mental health infrastructure, funding for university counseling services, and public campaigns promoting mental health awareness are essential components of a comprehensive recovery plan.

The protective role of social support underscores its critical importance for mental health. Public health strategies should focus on fostering peer networks and supportive environments within academic settings. This can be achieved through mentorship programs, peer support groups, and student-led initiatives designed to build connections among students. The positive association between engaging in activities with others and improved wellbeing suggests that promoting collective activities could mitigate loneliness and depression. Universities and community organizations should invest in facilitating safe, inclusive, and engaging social opportunities to encourage participation in group activities, both in-person and virtual. Moreover, differences in wellbeing outcomes by gender and international student status indicate the need for tailored interventions. Female students and long-term international students may require additional support, such as culturally sensitive counseling services, gender-specific mental health programs, and initiatives to integrate international students into campus life. The long-lasting effects of the pandemic on students' quality of life call for public health measures to strengthen resilience against future crises. This includes providing skills-based training in stress management, fostering emotional regulation, and offering resources to navigate challenging circumstances. Universities should collaborate with public health agencies to embed these initiatives in student health services.

By integrating these findings into public health planning and university policies, stakeholders can improve the wellbeing of college students and create more resilient systems to support young adults in both current and future challenges.

## Data Availability

The datasets presented in this study can be found in online repositories. The names of the repository/repositories and accession number(s) can be found at: https://osf.io/4h8vn/.
